# Evaluation of the Physicochemical Properties, Pharmacokinetics, and In Vitro Anticancer Effects of Docetaxel and Osthol Encapsulated in Methoxy Poly(ethylene glycol)-*b*-Poly(caprolactone) Polymeric Micelles

**DOI:** 10.3390/ijms22010231

**Published:** 2020-12-28

**Authors:** Min Jeong Jo, Yu Jin Lee, Chun-Woong Park, Youn Bok Chung, Jin-Seok Kim, Mi Kyeong Lee, Dae Hwan Shin

**Affiliations:** 1 College of Pharmacy, Chungbuk National University, Osongsaengmyeong 1-ro, Osong-eup, Heungdeok-gu, Cheongju 28160, Korea; jmj950912@gmail.com (M.J.J.); 5yujinli15@gmail.com (Y.J.L.); cwpark@chungbuk.ac.kr (C.-W.P.); chungyb@chungbuk.ac.kr (Y.B.C.); mklee@chungbuk.ac.kr (M.K.L.); 2 Drug Information Research Institute (DIRI), College of Pharmacy, Sookmyung Women’s University, Cheongpa-ro 47-gil 100, Yongsan-gu, Seoul 04310, Korea; jsk9574@sookmyung.ac.kr

**Keywords:** docetaxel, osthol, mPEG-*b*-PCL polymeric micelles, combination therapy, pharmacokinetics

## Abstract

Docetaxel (DTX), a taxane-based anticancer drug, and osthol (OTH), a coumarin-derivative compound, have shown anticancer effects against different types of cancers through various mechanisms. However, these drugs have low solubility in water and low oral bioavailability, and thus their clinical application is difficult. To overcome these problems, we encapsulated DTX and OTH in methoxy poly(ethylene glycol)-*b*-poly(caprolactone) (mPEG-*b*-PCL) and conducted studies in vitro and in vivo. We selected a 1:4 ratio as the optimal ratio of DTX and OTH, through combination index analysis in A549 cancer cells, and prepared micelles to evaluate the encapsulation efficiency, drug loading, particle size, and zeta potential. The in vitro drug-release profile showed that DTX/OTH-loaded mPEG-*b*-PCL micelles could slowly release DTX and OTH. In the clonogenic assay, DTX/OTH-loaded mPEG-*b*-PCL micelles showed 3.7 times higher inhibitory effect than the DTX/OTH solution. Pharmacokinetic studies demonstrated that micelles in combination with DTX and OTH exhibited increased area under curve and decreased clearance values, as compared with single micelles.

## 1. Introduction

Lung cancer is one of the leading causes of cancer-related deaths worldwide [1,2,3]. There are two types of lung cancer, non-small-cell lung cancer and small-cell lung cancer, with the former accounting for about 80–85% of lung cancer cases [4,5]. Several therapies, such as chemotherapy and molecular targeted therapy, have been developed for anticancer treatment [6,7,8]. The commonly used chemotherapeutic drugs include taxane-based drugs, such as paclitaxel and docetaxel, as well as doxorubicin and camptothecin [9]. However, these therapies are often associated with systemic toxicity, and low drug concentrations at the tumor site can lead to multiple drug resistance [10]. In addition, high-dose medications are required to kill the tumor, which can cause serious side effects in patients [11]. Therefore, there is a need to search for new chemotherapy and drug delivery systems with improved efficacy against lung cancer.

Docetaxel (DTX) is a taxane-based drug used to treat several types of solid tumors, such as breast, head and neck, stomach, prostate, and non-small-cell lung cancer [12,13]. DTX induces cell death by inhibiting microtubule degradation and preventing microtubule aggregation [14,15]. It is also known to induce phosphorylation of the tumor protein bcl-2, which blocks cell death [16]. However, the anticancer effect is often reduced due to drug resistance of DTX. Furthermore, its solubility in water is low, which limits its clinical application [17,18,19].

Osthol (OTH), a derivative of coumarin, is a natural ingredient found in a variety of plants, including *Cnidium monnieri*, *Angelica archangelica*, and *Angelica pubescens* [20]. Several studies have shown that OTH has antioxidant [21], anti-inflammatory [22], anti-osteoporosis [23], and anti-allergic effects [24] and exhibits various biological and pharmacological activities. In addition, OTH exhibits antitumor effects by inhibiting tumor cell growth and inducing apoptosis. Jiang et al. demonstrated that OTH is a selective antitumor agent against ovarian cancer and exerts its effects by inhibiting cell proliferation, cell migration, and cell invasion, as well as by inducing apoptosis and G2/M arrest [25]. Wang et al. demonstrated that, in osteosarcoma cells, OTH inhibits induced cell-cycle arrest and migration and invasion by modulating the PTEN/Akt pathway, to alter downstream molecular expression and activity [26]. Xu et al. showed that OTH inhibits proliferation and induces apoptosis in A549 lung cancer cells through an IAP family protein, and they demonstrated that OTH enhances the antitumor effect of embelin, resulting in a synergistic effect of the two drugs [27]. Yang et al. demonstrated that OTH has antitumor activity against gastric and breast cancer cells, which is due to cell cycle arrest in the G2/M phase and apoptosis. In addition, in this study, the combination of trastuzumab and OTH was shown to be much more potent in inhibiting gastric cancer cells than either drugs alone [28]. Thus, the combination of OTH and other conventional anticancer drugs was shown to have a better effect against cancer cells than single chemotherapeutic agent. Several papers have reported that the combination of DTX and a drug with a mechanism similar to OTH used as an inhibitor of Akt exhibits a synergistic effect. Sánchez et al. demonstrated that the combination of DTX and capsaicin induces potent AMPK activation and Akt/mTOR axe inhibition in PC3 prostate tumors [29]. Singh et al. showed that the combination of DTX and thymoquinone inhibited the PI3K/Akt pathway, which induces the death of prostate cancer cells [30]. Accordingly, we predicted that the combination of DTX and OTH used as an Akt inhibitor would effectively inhibit cancer cells and exhibit a synergistic effect. However, similar to DTX, OTH is insoluble in water, and hence it has limited bioavailability [31].

Solvents such as polysorbate 80 (Tween 80^®^), Cremophor EL^®^, ethanol (EtOH), and dimethylacetamide (DMA) are used to solubilize poorly soluble drugs in intravenous formulations. Taxotere^®^, a formulation in which docetaxel is solubilized by using a mixture of Tween 80^®^ and 13% EtOH as a solvent, exhibited side effects, such as acute hypersensitivity reactions and peripheral neuropathy, due to the toxicity of the solvent [32]. Tween 80^®^ also exhibits side effects, such as nonallergic anaphylaxis and rash, and has been associated with kidney and liver toxicity [33]. To replace these toxic solubilizing agents, in this study, we used micelles for the combination of DTX and OTH, using the mPEG-*b*-PCL polymer. Polymeric micelles are biocompatible, biodegradable, non-toxic, and exhibit improvement in blood flow at the treatment site, allowing for better accumulation of the drug at the tumor tissues, and thereby overcoming the effects of multiple-drug resistance [34,35]. Methoxy poly(ethylene glycol)-*b*-poly(caprolactone) (mPEG-*b*-PCL) is a self-assembled diblock copolymer with a hydrophilic shell (PEG) and a hydrophobic core (PCL). This polymer is characterized as being non-toxic, and its degradation products can be easily excreted and do not accumulate in vivo [36]. In addition, many studies have shown that mPEG-*b*-PCL increases the solubility of the drug, allows controlled and sustained release of the drug, and helps in maintaining better bioactivity of the drug [37,38]. Cho et al. demonstrated that mPEG-*b*-PCL micelles were effective in peritoneal tumors by enabling IP combination drug delivery of paclitaxel, cyclopamine, and gossypol and simultaneous sustained release of the three drugs in vitro [39].

In this study, we investigated the synergistic effect of DTX and OTH encapsulated in micelles, using mPEG-*b*-PCL. The physicochemical properties, in vitro release, in vitro cytotoxicity, and pharmacokinetic profile of the micelles were evaluated. We believe that the findings from this study will be of great help in facilitating clinical research and application of the combination of DTX and OTH for the treatment of lung cancer.

## 2. Results

### 2.1. Evaluation of Synergistic Effects of DTX and OTH

The potential synergistic effects of DTX and OTH were determined in A549 cells by measuring the 50% inhibition concentration (IC_50_) values of DTX and OTH at different ratios and analyzing the combination index (CI) values. Table 1 shows the IC_50_ values of DTX and OTH at each ratio, indicating that the cell proliferation inhibitory effect varied depending on the combination ratio. In addition, CI values were calculated at the ratios 11:1, 4:1, 2:1, 1:1, 1:2, 1:4, and 1:11, to determine the optimal ratio of DTX and OTH. Based on the results of the CI analysis, values of 1.85 and 2.97 were obtained at the ratio of 11:1 and 4:1, respectively, and these values indicated antagonism because the CI was greater than 1. In contrast, when the ratio was 2:1, 1:1, 1:2, 1:4, and 1:11, the CI was less than 1, indicating synergism. Therefore, the ratio showing synergism was selected as the optimal ratio and used for the subsequent preparation of drug-loaded mPEG-*b*-PCL micelles.

### 2.2. Preparation and Characterization of DTX/OTH-Loaded mPEG-b-PCL Micelles

**Figure 1:**
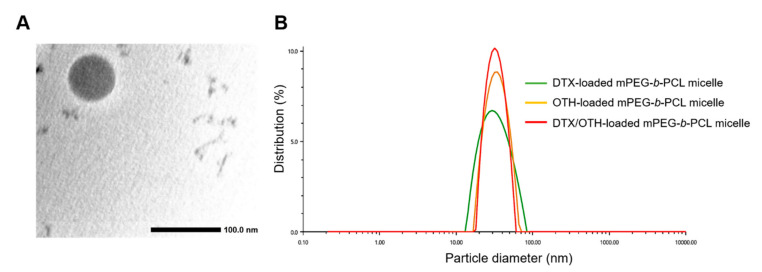
Transmission electron microscopy (TEM) image of docetaxel (DTX) and osthol (OTH)-loaded mPEG-*b*-PCL micelles (**A**). Representative size distribution analysis of DTX-loaded mPEG-*b*-PCL micelles, OTH-loaded mPEG-*b*-PCL micelles, and DTX/OTH-loaded mPEG-*b*-PCL micelles (**B**).

Table 2 shows the results related to the encapsulation efficiency (EE, %), drug loading (DL, %), particle diameter, poly-dispersity index (PDI), and zeta potential of mPEG-*b*-PCL micelles loaded with DTX and OTH. It was observed that at a ratio of 2:1, the EE (%) value of OTH was 82.2–109%, which was higher than other ratios, but the EE (%) value of DTX was 54.0–67.4%, which was significantly lower than other ratios. For the remaining ratios (1:2, 1:4, 1:11), DTX showed an EE (%) value within the range of 69.1–76.2%, and OTH showed a similar overall pattern, with an EE (%) value of 74.5–82.3%. Furthermore, the overall drug-loading value decreased as the amount of polymer increased. Therefore, we selected the 1:4 ratio, using 50 mg of the polymer as the optimal ratio when considering various factors, and the formulation that doubled the amount of the drug was selected as the final formulation and used in further in vitro and in vivo studies. In addition, transmission electron microscopy (TEM) images showed that mPEG-*b*-PCL micelles equipped with a 1:4 ratio of DTX/OTH indicated a uniform spherical shape (Figure 1A), and the size distribution graph showed that the size of single and combined micelles was less than 100 nm (Figure 1B).

### 2.3. In Vitro Drug Release Assay

**Figure 2:**
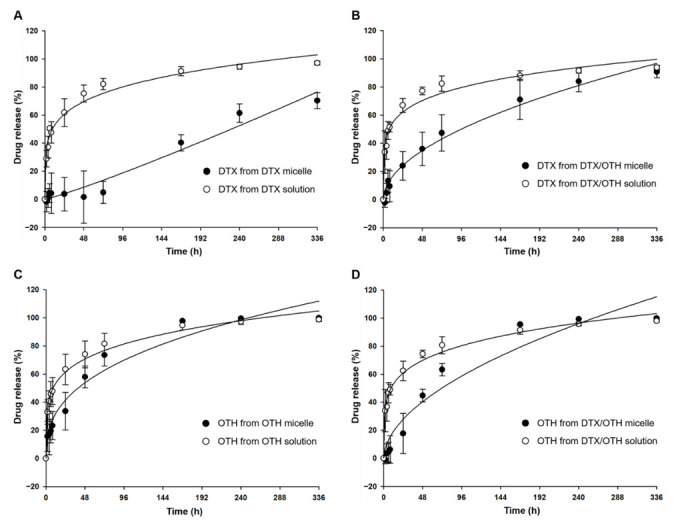
Single and combined drug-release profiles of docetaxel (DTX) and osthol (OTH). (**A**) DTX release in DTX-loaded mPEG-*b*-PCL micelles and DTX solution, (**B**) DTX release in DTX/OTH-loaded mPEG-*b*-PCL micelles and DTX/OTH solution, (**C**) OTH release in OTH-loaded mPEG-*b*-PCL micelles and OTH solution, and (**D**) OTH release in DTX/OTH-loaded mPEG-*b*-PCL micelles and DTX/OTH solution.

Single and combined drug-release profiles of DTX and OTH are shown in Figure 2. As shown in Figure 2A, after 6 h, the release rate of DTX was 4.4% in DTX-loaded mPEG-*b*-PCL micelles, and the release rate of DTX was 50.5% in DTX solution (*p* < 0.05). At 336 h, 96.9% of DTX was released from the DTX solution, while 70.3% of DTX was released from the DTX-loaded mPEG-*b*-PCL micelle, indicating that the release of DTX from mPEG-*b*-PCL micelles was slower than that from the solution (*p* < 0.05). Figure 2B shows that, during the first 72 h, the DTX release rate of the DTX/OTH solution was 82.3%, and that of DTX/OTH-loaded mPEG-*b*-PCL micelles was 47.4%, indicating that the drug release from the micelles was slower than that from the solution (*p* < 0.05). In Figure 2C, the release rate of OTH in OTH-loaded mPEG-*b*-PCL micelles during the first 72 h was 73.5%, and the release rate of OTH in OTH solution was 81.5%. Similarly, Figure 2D shows that, during the first 72 h, the release rate of OTH in the DTX/OTH-loaded mPEG-*b*-PCL micelles was 63.2%, and the release rate of OTH in the DTX/OTH solution was 80.6% (*p* < 0.05), which indicates that the drug release from micelles was slower than for the solution.

### 2.4. Cytotoxicity of DTX/OTH-Loaded mPEG-b-PCL Micelles in A549 Cells

**Figure 3:**
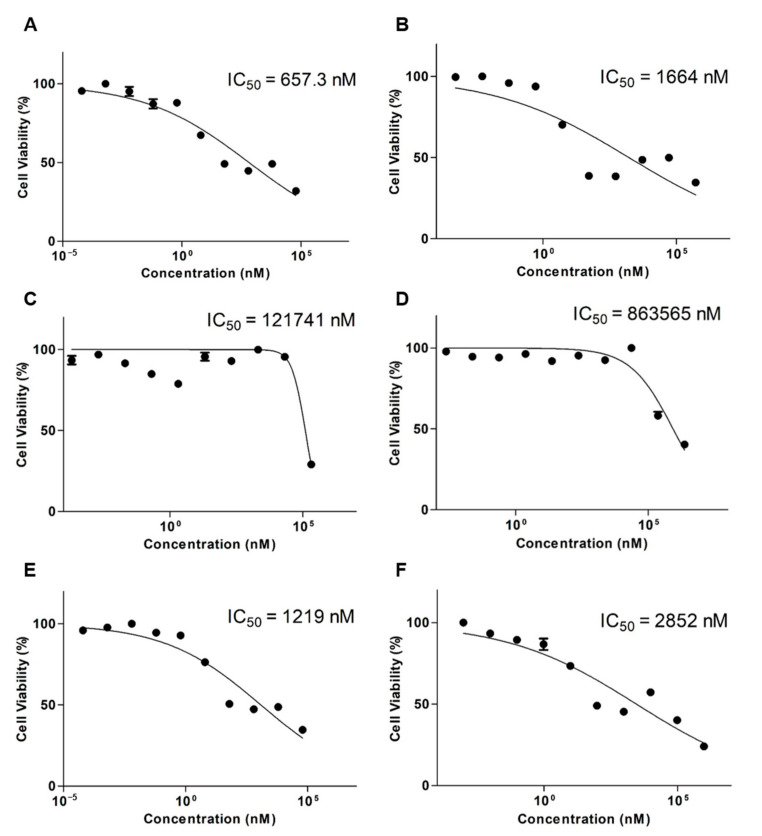
In vitro cytotoxicity assay results after the treatment of A549 cells with (**A**) docetaxel (DTX) free drug, (**B**) DTX-loaded mPEG-*b*-PCL micelles, (**C**) osthol (OTH) free drug, (**D**) OTH-loaded mPEG-*b*-PCL micelles, (**E**) DTX/OTH free drug, and (**F**) DTX/OTH-loaded mPEG-*b*-PCL micelles.

Figure 3 shows the cytotoxicity results of DTX, OTH, and DTX, as well as OTH combinations with the final ratio of DTX and OTH of 1:4. The IC_50_ value of the DTX free drug was 657.3 nM (Figure 3A), and the IC_50_ value of DTX-loaded mPEG-*b*-PCL was 1664 nM (Figure 3B). In addition, the IC_50_ value of OTH free drug was 121,741 nM (Figure 3C), and the IC_50_ value of OTH-loaded mPEG-*b*-PCL was 863,565 nM (Figure 3D). Finally, the IC_50_ value of DTX/OTH free drug was 1219 nM (Figure 3E), and the IC_50_ value of DTX/OTH-loaded mPEG-*b*-PCL was 2852 nM (Figure 3F).

### 2.5. Clonogenic Assay

**Figure 4:**
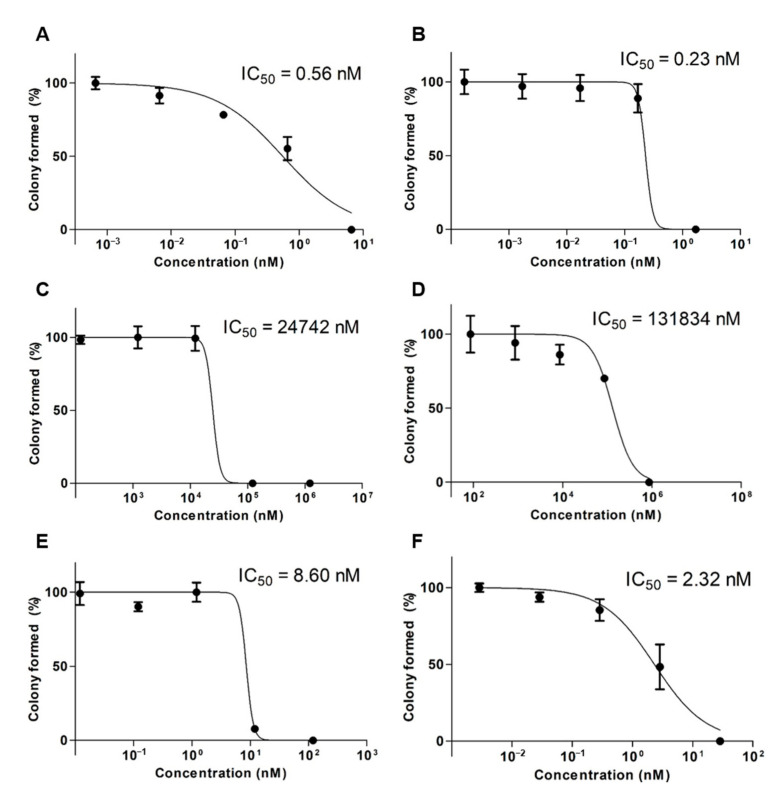
In vitro clonogenic assay results after the treatment of A549 cells with (**A**) docetaxel (DTX) free drug, (**B**) DTX-loaded mPEG-*b*-PCL micelles, (**C**) osthol (OTH) free drug, (**D**) OTH-loaded mPEG-*b*-PCL micelles, (**E**) DTX/OTH free drug, and (**F**) DTX/OTH-loaded mPEG-*b*-PCL micelles.

To assess cytotoxicity over a long period of time, a clonogenic assay was performed by calculating the IC_50_ values at different concentrations (Figure 4). When comparing the single formulation of DTX and OTH, there was little or no colony inhibitory effect, but in the combined formulation, DTX/OTH-loaded mPEG-*b*-PCL micelles inhibited colony formation that was 3.7 times higher than that of the DTX/OTH free drug.

### 2.6. In Vivo Pharmacokinetic Study

**Figure 5:**
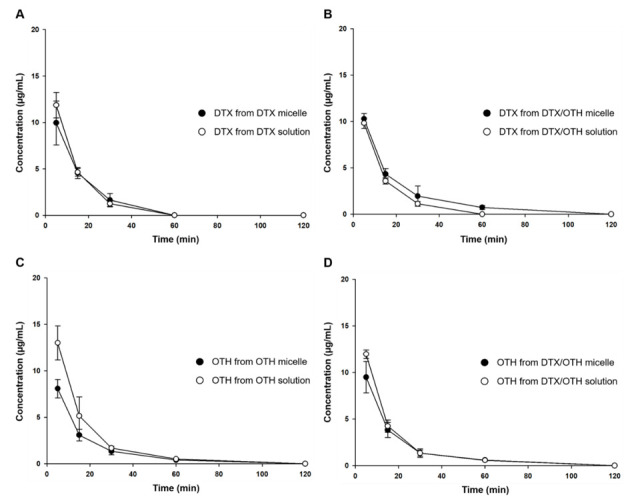
Plasma concentration–time profiles after intravenous injection of docetaxel (DTX) and osthol (OTH). (**A**) DTX profile in DTX-loaded mPEG-*b*-PCL micelles and DTX solution, (**B**) DTX profile in DTX/OTH-loaded mPEG-*b*-PCL micelles and DTX/OTH solution, (**C**) OTH profile in OTH-loaded mPEG-*b*-PCL micelles and OTH solution, and (**D**) OTH profile in DTX/OTH-loaded mPEG-*b*-PCL micelles and DTX/OTH solution.

Plasma concentration–time profiles and pharmacokinetic parameters for single and combination formulations of DTX and OTH are shown in Figure 5 and Table 3. As shown in Figure 5A, the plasma concentration of DTX in the single formulation was detected up to 30 min in both micelles and the solution, and after 1 h, the concentration was not detected below the limit of detection (LOD). Figure 5B shows that in the formulation using DTX and OTH in combination, DTX plasma concentration was detected up to 30 min in the solution, whereas, it was detected up to 1 h in micelles. In addition, DTX solution had a slightly higher area under curve (AUC) value than micelles in the single formulation, but there was no significant difference (Table 3). In contrast, in the combination formulation, the AUC value of DTX of the combined micelles was 1.3 times higher than that of the combination solution. In Figure 5C, the plasma concentration of OTH in the single formulation was detected up to 1 h in both micelles and solution, and after 2 h, it was not detected below LOD. Figure 5D shows that in the plasma concentration of OTH in the combination formulation, both micelles and solutions were detected up to 1 h, similar to the single formulation. The parameters of OTH showed a significant difference in the AUC value of OTH in the single formulation solution and micelles (*p* < 0.05), and the AUC value of OTH in the combined solution was slightly higher than that of the combined micelles, but there was no significant difference.

### 2.7. Biodistribution Study

**Figure 6:**
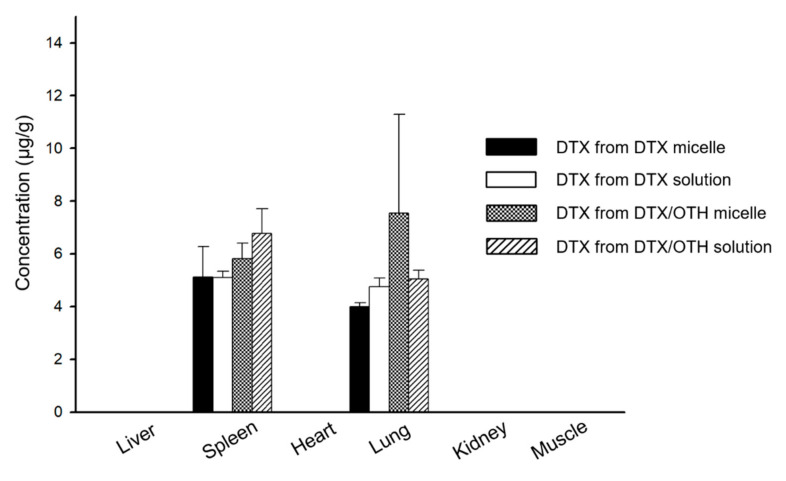
Biodistribution of docetaxel (DTX) in single and combined formulations, at 8 h after intravenous injection, in each tissue.

Figure 6 shows a graph of drug distribution in major organs, at 8 h after intravenous injection. DTX was detected in some organs, but OTH was not detected in all organs. DTX was found to be distributed in the lungs and spleen, both in solution and micelles. Among the two organs, the DTX concentration of combined micelles was the highest in the lungs, while that in the spleen was 1.3 times lower than that in the lungs. In addition, compared to single micelles, higher amounts of DTX were detected in both the spleen and lung for combined micelles (no significant difference).

## 3. Discussion

DTX and OTH are widely used for the treatment of various types of cancers and have a variety of biological and pharmacological activities. However, low solubility in water and low oral bioavailability have limited their clinical application [40,41]. To overcome these disadvantages, we selected micelles for the solubilization of DTX and OTH and expected the synergistic effect of the two drugs. Before encapsulating the two drugs in micelles, we evaluated the CI value to find the optimal ratio at which the synergistic effect of DTX and OTH could be achieved. Among the four ratios evaluated (1:2, 2:1, 1:4, and 1:11), the ratio showing the smallest CI value (CI value less than 1) was selected and used for micelle formulation. Micelles were prepared at each ratio, and the physicochemical properties, including EE (%), particle size, PDI, and zeta potential, were evaluated. Finally, a 1:4 ratio was chosen with an optimal formulation with low CI values of 0.38, high EE (%) and DL (%), appropriate particle size, and low PDI values. In addition, we hypothesized that by doubling the amount of the drug, a higher plasma concentration could be achieved in the subsequent in vivo study. Dynamic light scattering (DLS) analysis, TEM, and size-distribution graph results showed that the size of micelles was less than 100 nm. These results show that micelles with a particle size of less than 100 nm may allow very low absorption by the reticuloendothelial system (RES) and their accumulation into tumors through enhanced permeability and retention (EPR) effects [42]. Results from the in vitro release profiles of both DTX and OTH showed that drug release rate from the micelles was slower than those from the solution. These results suggest that the drug is trapped in the micelles, limiting the rapid release of the drug. It has also been shown that hydrophobic drug-encapsulated micelle carriers increase the solubility of drug and enable slower release of the drug [43]. In an in vitro cytotoxicity assay, the cytotoxic results of DTX and OTH were confirmed at 48 h. The IC_50_ values of both DTX and OTH were higher in micelle formulations than in the free drugs, which can be explained in relation to the release profile. The 48 h release rate of DTX in DTX/OTH solution was 77.2%, whereas the release rate of DTX in DTX/OTH-loaded mPEG-*b*-PCL micelles was only 36%. Similarly, the 48 h release rate of OTH in DTX/OTH solution was 74.4%, while that of the DTX/OTH-loaded mPEG-*b*-PCL micelles was only 44.8%. Based on these findings, it was evident that the cytotoxic effect of the drugs in the micelles was lower than the free drug due to the slow release of the drug in the micelles over 48 h. In addition, while free drugs can enter the cell membrane by passive diffusion, micelles have a slower endocytosis process, and regulation of drug release may delay the process of cancer cell death [44]. In the clonogenic assay, the rate of colony inhibition of DTX and OTH over two weeks was confirmed. In the single formulation of DTX and OTH, the colony inhibition rate of micelles was slightly higher or lower than that of the free drug, but when used in combination, the colony inhibition rate of micelles was 3.7 times higher than that of the free drug. This indicated that the combination therapy was more effective in suppressing cancer cells than therapy with a single drug. In the in vivo pharmacokinetic study, single and combined formulations of DTX and OTH were analyzed, using plasma-time concentration graphs and pharmacokinetic parameters. The single formulation DTX solution and micelles had AUC values of 222 and 205 min·µg·mL^−1^, respectively, and the single formulation OTH solution and micelles showed AUC values of 274 and 177 min·µg·mL^−1^, respectively, indicating that the solution had a higher AUC value than micelles. This phenomenon is predicted to lower AUC, as DTX and OTH in micelles are rapidly distributed from the plasma to tissues after intravenous administration of the drug. Moreover, the reason that the solution has a higher AUC than the micelle may be due to the high plasma accumulation of the drug due to the significant interaction between the drug and the hydrophobic group of the fatty acid chain in the solution group. On the other hand, studies have shown that the inner core of micelles with weak hydrophobic interactions cannot capture the drug, which is thought to lower the AUC of the drug in the plasma [45,46,47,48,49]. However, a comparison of single micelles and combined micelles showed that using DTX and OTH together increased the AUC value and decreased the CL_t_ value. These results indicate that the bioavailability is improved when DTX and OTH are used in combination than when using a single formulation. In the biodistribution study, DTX accumulated in the lungs and spleen, which can be seen as a result of drug absorption by the RES organ [50]. These results can be beneficial for the treatment of lung cancer, as micelles are detained in the lungs, and the drug can be continuously released [49].

## 4. Materials and Methods

### 4.1. Materials and Reagents

The mPEG-*b*-PCL (Mw~2000:2000Da) was purchased from Polyscitech. (West Lafayette, IN, USA). DTX and genistein were purchased from LC Laboratories (Woburn, MA, USA). OTH was obtained from Chungbuk National University, Cheongju, Korea and identified by Professor Mi Kyeong Lee, who specializes in natural products. Ethanol (EtOH) and acetonitrile (ACN) were purchased from Fisher Scientific Ltd. (Waltham, MA, USA). Distilled water (DW) was purchased from Tedia (Fairfield, OH, USA). Methanol (MeOH) was purchased from Honeywell Burdick and Jackson (Ulsan, Korea). Polysorbate 80 (Tween 80^®^) was purchased from Sigma-Aldrich Corp. (St. Louis, MO, USA). All other chemicals were of analytical reagent grade or better.

### 4.2. Methods

#### 4.2.1. Cell Line and Cell Culture

A549 cells were purchased from the American Type Culture Collection (Manassas, VA, USA). Roswell Park Memorial Institute medium (RPMI 1640), Dulbecco’s modified Eagle’s medium, Dulbecco’s phosphate-buffered saline (DPBS), and trypsin were purchased from Corning Inc. (Corning, NY, USA). Thiazolyl blue tetrazolium bromide (MTT) was purchased from Sigma-Aldrich Corp. (St. Louis, MO, USA). The cells were cultured in RPMI medium supplemented with 1% (*w/v*) streptomycin/penicillin and 10% (*v/v*) fetal bovine serum (FBS), in a humidified 5% CO_2_ atmosphere, at 37 °C.

#### 4.2.2. High-Performance Liquid Chromatography (HPLC) Analysis

The concentration of DTX and OTH in the samples was analyzed by using a Waters HPLC system (Milford, Massachusetts, USA) consisting of a 2695 separation module and a 2996 photodiode array detector. A Fortis C18 chromatography column (5 µm, 4.6 × 250 mm) was used for the analysis, and the column was kept at 30 °C. DTX, OTH, and genistein (internal standard, IS) were eluted in isocratic mode; the injection volume was 10 µL, and the flow rate of the mobile phase consisting of ACN/water (70:30, *v/v*) was 1.0 mL/min. DTX, OTH, and IS were detected at the wavelengths of 230, 320, and 259 nm, respectively. The retention times of DTX, OTH, and IS were 4.7, 8.6, and 3.5 min, respectively. The concentration of each drug was calculated by comparing the peak areas with the calibration curve.

#### 4.2.3. In Vitro Cytotoxicity Assay

The cytotoxicity of DTX/OTH-loaded mPEG-*b*-PCL micelles on A549 cells was evaluated by the MTT assay [51]. A549 cells were seeded in 96-well plates, at a density of 5000 cells per well and incubated at 37 °C for 24 h. After 24 h, the medium was removed, and the cells were treated with free DTX, free OTH, free DTX/OTH, DTX-loaded mPEG-*b*-PCL micelles, OTH-loaded mPEG-*b*-PCL micelles, or DTX/OTH-loaded mPEG-*b*-PCL micelles according to each concentration (*n* = 6). Fresh medium containing no drug was used as a control. The cells were incubated at 37 °C for 48 h. After 48 h, the medium was removed, and 100 μL of MTT solution (0.5 mg/mL) was added, followed by incubation for 4 h. Then, the medium was removed, and 100 μL of dimethyl sulfoxide was added, and the plate was shaken for 10 min at 200 rpm. Absorbance was measured at 540 nm, using a microplate reader (Spectra Max ID3, Molecular Devices, San Jose, CA, USA). All data processing was analyzed by using GraphPad Prism 5 software (GraphPad Software, La Jolla, CA, USA).

#### 4.2.4. Combination Index (CI) Analysis

CI analysis was performed according to Chou’s method to assess drug interaction between drugs [52]. The CI values of DTX and OTH were calculated by using the following equation:

Combination index (CI) = (D)_1_/(D_x_)_1_ + (D)_2_/(D_x_)_2_

where is (D_x_)_1_ and (D_x_)_2_ are the inhibitory concentrations of drug 1 and drug 2, respectively. (D)_1_ and (D)_2_ are the inhibitory concentrations of each drug in the combination. CI > 1 indicates antagonism, CI < 1 indicates synergism, and CI = 1 indicates an additive effect.

#### 4.2.5. Clonogenic Assay

A549 cells were seeded in 6-well plates, at a density of 200 cells per well, and incubated at 37 °C for 24 h. After confirming cell adhesion, the cells were treated with free DTX, free OTH, free DTX/OTH, DTX-loaded mPEG-*b*-PCL micelles, OTH-loaded mPEG-*b*-PCL micelles, or DTX/OTH-loaded mPEG-*b*-PCL micelles. After two weeks, the medium was removed, and 1 mL of crystal violet (0.5% *w/v*) was added to each well. After incubation for 30 min, the crystal violet was rinsed with clean water, and the number of colonies was measured. The colony formation percentage was calculated by using the equation below:

Colony formation (% of control) = Number of colonies after treatment/Number of colonies of control (PBS) × 100

#### 4.2.6. Preparation of DTX- and OTH-Loaded Polymeric Micelles

DTX- and OTH-loaded mPEG-*b*-PCL micelles were prepared by using the thin-film hydration method [53]. Briefly, various ratios of DTX and OTH were mixed with mPEG-*b*-PCL, dissolved in 1 mL of ACN, and placed in a round-bottomed flask. The DTX and OTH-polymer mixture was evaporated under vacuum, using a EYELA^®^ rotary evaporator (Bohemia, NY, USA), in a water bath, for 10 min, maintained at 60 °C. After a thin film was formed, 1 mL of DW was added, and the micelle solution obtained by hydration for 30 min was centrifuged at 16,600× *g* for 5 min (Hanil Science Inc., Gimpo, Korea). Then, the supernatant of the centrifuged micelles was obtained and filtered through a 0.2 μm filter, and the physicochemical properties of the micelles were confirmed.

#### 4.2.7. Physicochemical Characterization of Micelles

The particle size and zeta potential of DTX/OTH-loaded mPEG-*b*-PCL micelles were measured by using a dynamic light scattering (DLS) device (Litesizer 500, Anton Paar, Graz, Austria). Each sample was diluted 10 times prior to measurement. The drug content of mPEG-*b*-PCL micelles was analyzed by HPLC, under the conditions mentioned in Section 4.2.2. Then, 20 μL of drug-loaded mPEG-*b*-PCL micelle solution was dissolved in 180 μL of ACN. The encapsulation efficiency (EE, %) and drug loading (DL, %) of DTX and OTH were calculated by using the equation below [54,55,56]:

DL% = Weight of drug in micelles/weight of feeding drug and polymer × 100

EE% = Weight of drug in micelles/weight of feeding drug × 100

The morphology of DTX/OTH-loaded mPEG-*b*-PCL micelles was observed by using a JEM-2100 transmission electron microscope (TEM, JEOL Ltd., Tokyo, Japan). In the sample preparation procedure for TEM measurement, the diluted micelle solution was dropped on 200-mesh formvar-coated copper grids and dried in a dry oven at 60 °C for 12 h. The results of each sample analysis are presented as the mean ± standard deviation of three separate experiments.

#### 4.2.8. In Vitro Drug Release Assay

The in vitro release profile of DTX and OTH in the micelles was evaluated by using a dialysis method in phosphate-buffered saline (PBS, pH 7.4) [57]. Briefly, DTX solution, OTH solution, DTX/OTH solution, DTX-loaded mPEG-*b*-PCL, OTH-loaded mPEG-*b*-PCL micelles, and DTX/OTH-loaded mPEG-*b*-PCL micelles were placed in a dialysis membrane (MWCO 20 kD) of 2.0 L release medium, stirred at 200 rpm, at 37 °C. The solution was prepared in a ratio of 25%, 10%, and 65% of Tween 80^®^, EtOH, and DW, respectively, using the same solvent (Taxotere^®^) as the control. In both in vitro release experiments and pharmacokinetic experiments, DTX solution and micelles were used at a concentration of 4 mg/mL, and OTH was used at a concentration of 5 mg/mL. At each sampling time point (0, 2, 4, 6, 8, 24, 48, 72, 168, 240, and 336 h), 20 μL of each sample was withdrawn, and then each sample was diluted 10 times with ACN, and the DTX and OTH concentrations were measured by HPLC. The PBS release medium was replaced with fresh media at 8, 72, 144, 216, and 288 h. All experiments were conducted three times.

#### 4.2.9. Pharmacokinetic Study

Sprague-Dawley rats (male, 7 weeks old) were obtained from Orient Bio Inc. (Seongnam, Korea) for pharmacokinetic studies. Animal experiments were approved by the Institutional Animal Care and Use Committee (IACUC) of Chungbuk National University (CBNUR-1405-20, 23 July 2020). All rats were maintained in well-ventilated cages and fed with adequate amounts of water and food. The rats were divided into six groups: DTX solution, OTH solution, DTX/OTH solution, DTX-loaded mPEG-*b*-PCL micelles, OTH-loaded mPEG-*b*-PCL micelles, and DTX/OTH-loaded mPEG-*b*-PCL micelles. The micelles were injected with a size of 30 nm, and the solution was used with the same solvent as mentioned in Section 4.2.8. Each sample was injected intravenously, and DTX was administered at a dose of 10 mg/kg and OTH 12 mg/kg. At predetermined time intervals (5, 15, 30, 60, 120, 240, and 480 min), 500 µL of blood was collected from the retro-orbital plexus and placed in a heparin tube. Then, the samples were immediately separated by centrifugation at 16,600× *g* for 5 min and stored at −70 °C, until analysis. The pharmacokinetic parameters, including initial blood concentration (C_0_), area under the concentration–time curve (AUC), volume of distribution (V_d_), and total clearance (CL_t_) were calculated by using Sigma Plot 10.0 (Systat Software, San Jose, CA, USA). All experiments were repeated three times.

#### 4.2.10. Biodistribution Study

A biodistribution study was performed to evaluate the tissue distribution of DTX and OTH formulations after pharmacokinetic studies were performed. The rats were euthanized, using CO_2_ gas 8 h after administration. Tissue samples from the liver, spleen, heart, lungs, kidneys, and muscles were collected. Each sample was washed with saline and wiped with a paper towel and stored at –70 °C, until analysis.

#### 4.2.11. Biological Sample Pretreatment for HPLC Analysis

Plasma samples that were frozen before analysis were thawed at ambient temperature. To a 200 µL of plasma sample, 400 µL of MeOH and 50 µL of IS were added, followed by centrifugation at 16,600× *g* for 5 min. The supernatant was filtered through a 0.2 µm cellulose filter and analyzed by HPLC [49]. Biodistribution samples were evaluated using the homogenization method [58]. Briefly, each tissue sample (liver, spleen, heart, lungs, kidneys, and muscles) was homogenized with a Teflon pestle into a glass Potter–Elvehjem-type homogenizer (Ultra Turrax T-25; IKAWorks Inc., Staufen, Germany). Then, 200 μL of each tissue sample was collected, MeOH and IS were added and pretreated in the same manner as described above, and the concentrations of DTX and OTH were analyzed by HPLC, as mentioned in Section 4.2.2.

#### 4.2.12. Statistical Analysis

All experiments were performed in triplicate, and all data values are expressed as mean ± SD. Statistical analysis was performed by using ANOVA of GraphPad Prism v 5.0 (GraphPad Software, La Jolla, CA, USA), and values of *p* < 0.05 are considered significant.

## 5. Conclusions

In conclusion, we prepared polymeric micelles for solubilization of DTX and OTH and investigated the synergistic effects of the two drugs. In vitro studies showed that DTX/OTH-loaded mPEG-*b*-PCL micelles were released more slowly than DTX/OTH solutions, and showed higher colony inhibition rate than DTX/OTH-free drugs. In pharmacokinetic studies, micelles in combination with DTX and OTH showed increased AUC than single micelles and had improved bioavailability. Therefore, the synergistic effect of DTX and OTH offers a promising treatment strategy for lung cancer and will be of value for future preclinical studies.

## Figures and Tables

**Table 1:** CI values of various ratios of docetaxel (DTX) and osthol (OTH).

DTX:OTH (Molar Ratio)	IC_50_ (nM)		CI Value
	DTX	OTH	
11:1	1214 ± 705	110 ± 64.1	1.85
4:1	1947 ± 550	487 ± 137	2.97
2:1	131 ± 60.9	65.5 ± 30.5	0.20
1:1	404 ± 206	404 ± 206	0.62
1:2	343 ± 278	686 ± 557	0.53
1:4	244 ± 101	975 ± 402	0.38
1:11	68.8 ± 23.1	757 ± 232	0.11

**Table 2:** Characteristics of docetaxel (DTX) and osthol (OTH)-loaded mPEG-*b*-PCL micelles (*n* = 3, mean ± SD).

Formulation	Polymer Amount Used (mg)	DTX Amount Used (mg)	OTH Amount Used (mg)	DTX Encapsulation Efficiency (EE %)	OTH Encapsulation Efficiency (EE %)	DTX Drug Loading (DL %)	OTH Drug Loading (DL %)	Particle Size (nm)	Poly-Dispersity Index (PDI)	Zeta Potential (mV)
DTX:OTH 1:2	50	4	2.4	73.4 ± 9.81	80.8 ± 12.6	5.43 ± 0.73	3.70 ± 0.58	30.9 ± 0.85	0.13 ± 0.65	0.97 ± 0.21
	100	4	2.4	76.2 ± 7.27	82.3 ± 10.4	2.93 ± 0.28	1.93 ± 0.24	30.9 ± 0.99	0.16 ± 4.00	2.17 ± 1.05
	150	4	2.4	69.6 ± 5.72	77.3 ± 11.8	1.81 ± 0.15	1.22 ± 0.19	30.1 ± 1.06	0.08 ± 2.31	2.40 ± 1.21
DTX:OTH 2:1	50	6.62	1.0	54.0 ± 18.1	109 ± 4.16	6.31 ± 2.12	2.14 ± 0.08	31.3 ± 1.52	0.12 ± 7.75	−1.03 ± 2.12
	100	6.62	1.0	65.4 ± 5.31	82.2 ± 11.7	4.06 ± 0.33	0.81 ± 0.12	31.4 ± 2.22	0.12 ± 7.54	1.73 ± 0.76
	150	6.62	1.0	67.4 ± 0.79	95.8 ± 32.6	2.85 ± 0.03	0.63 ± 0.22	31.1 ± 0.97	0.12 ± 4.04	6.07 ± 2.37
DTX:OTH 1:4	50	3	3.6	72.2 ± 10.8	77.2 ± 11.8	4.09 ± 0.61	5.19 ± 0.80	31.4 ± 0.20	0.08 ± 0.38	0.53 ± 1.46
	100	3	3.6	70.5 ± 5.01	78.1 ± 5.93	2.05 ± 0.15	2.72 ± 0.21	31.6 ± 0.97	0.10 ± 1.14	1.37 ± 2.80
	150	3	3.6	74.2 ± 8.55	80.6 ± 1.95	1.45 ± 0.17	1.89 ± 0.05	31.5 ± 1.29	0.13 ± 0.82	4.03 ± 2.32
	50	6	7.2	72.2 ± 4.53	78.6 ± 2.38	7.74 ± 0.49	9.90 ± 0.30	33.3 ± 0.28	0.09 ± 0.67	−0.60 ± 0.78
	150	6	7.2	69.1 ± 4.27	77.7 ± 3.47	2.66 ± 0.16	3.56 ± 0.16	33.5 ± 2.25	0.17 ± 5.55	2.67 ± 0.40
DTX:OTH 1:11	50	2	6.6	74.1 ± 5.25	78.9 ± 1.68	2.85 ± 0.20	9.20 ± 0.20	32.6 ± 0.53	0.11 ± 2.35	−0.47 ± 0.70
	100	2	6.6	70.5 ± 3.11	76.8 ± 5.03	1.38 ± 0.06	4.75 ± 0.31	32.4 ± 0.36	0.10 ± 2.81	3.47 ± 0.96
	150	2	6.6	70.8 ± 5.44	74.5 ± 5.86	0.93 ± 0.07	3.14 ± 0.25	32.2 ± 1.00	0.16 ± 3.59	4.17 ± 1.03

EE, encapsulation efficiency; DL, drug loading; PDI, poly-dispersity index.

**Table 3:** Pharmacokinetic parameters after intravenous injection of docetaxel (DTX) and osthol (OTH).

Parameters	DTX Solution	DTX Micelle	OTH Solution	OTH Micelle	DTX in Combination Solution	DTX in Combination Micelle	OTH in Combination Solution	OTH in Combination Micelle
Dose (µg·kg^−1^)	10,000	10,000	12,000	12,000	10,000	10,000	12,000	12,000
AUC (min·µg·mL^−1^)	222 ± 16.6	205 ± 43.3	274 ± 41.6	177 ± 23.5	183 ± 11.7	244 ± 42.4	246 ± 7.3	212 ± 24.2
CL_t_ (mL·kg^−1^·min)	45.2 ± 3.44	50.1 ± 9.47	44.6 ± 7.37	68.5 ± 9.3	54.8 ± 3.65	41.8 ± 7.4	48.7 ± 1.45	57.1 ± 6.21

^a^ AUC, area under the curve; ^c^ CL_t_, total clearance.

## Data Availability

All the data is actually available and the article contains the data.

## Abbreviations

DTX	Docetaxel
OTH	Osthol
mPEG-*b*-PCL	Methoxy poly(ethylene glycol)-*b*-poly(caprolactone)
EE	Encapsulation efficiency
DL	Drug loading
CI	Combination index
LOD	Limit of detection
PDI	Poly-dispersity index
RES	Reticuloendothelial system
